# Remote Supratentorial Subdural Hematoma Following Craniectomy and Evacuation of Hypertensive Cerebellar Hematoma

**DOI:** 10.7759/cureus.6977

**Published:** 2020-02-13

**Authors:** Ravi R Pradhan, Gentle S Shrestha, Gopal Sedain

**Affiliations:** 1 Internal Medicine, Tribhuvan University Institute of Medicine, Kathmandu, NPL; 2 Critical Care, Tribhuvan University Teaching Hospital, Institute of Medicine, Kathmandu, NPL; 3 Neurosurgery, Tribhuvan University Institute of Medicine, Kathmandu, NPL

**Keywords:** cerebellar hematoma, decompressive craniectomy, remote acute subdural hematoma

## Abstract

Remote acute subdural hematoma following a decompressive craniotomy or craniectomy is a rare phenomenon. Only few cases of postoperative contralateral acute subdural hematomas have been reported in the literature review till date. This case report details a case of a 32-year-old hypertensive male who presented with severe headache, multiple episodes of vomiting, slurring of speech, nystagmus and ataxic gait for one day. Computed tomography (CT) scan of head revealed a right sided cerebellar hemorrhage with effacement of fourth ventricle and upstream hydrocephalus. A right suboccipital craniectomy and hematoma evacuation were performed. A repeat CT scan of head was done at six hours post surgery; which revealed a contralateral (left-sided) subdural hematoma involving the fronto-parieto-temporal region. The patient improved following conservative management.

Contralateral acute subdural hematoma following evacuation of hematoma is a rare, but a potentially life-threatening complication; therefore, we should try to detect such contralateral hematoma and prevent clinical deterioration.

## Introduction

Decompressive craniotomy or craniectomy is performed if there is no response to medical therapy in the increased intracranial pressure state. However, many complications may occur following the procedure [1,2]. Remote acute subdural hematoma (ASDH) following a decompressive craniotomy or craniectomy is a rare phenomenon [3,4]. Patients with ASDH suffer a high mortality and morbidity, despite modern surgical, medical and technological advances [4,5]. If unrecognized, it can have devastating consequences. Bilateral surgery after decompressive craniectomy is rarely needed (1%-4% of the cases), so its clinical features, outcomes and management are not well understood [6,7]. We report a case of contralateral remote supratentorial ASDH occurring after hematoma evacuation in a patient with hypertensive cerebellar hemorrhage.

## Case presentation

A 32-year-old male from Kathmandu presented to our center with headache, multiple episodes of vomiting, slurring of speech and tendency to fall on the right side while walking for one day. Headache began suddenly in the bilateral occipital region, was continuous and severe. The patient had a history of hypertension; however; he was non-compliant to the medication. He was a known smoker and drank alcohol regularly. There was no history of head trauma prior to the incident. The patient had family history of hypertension in his father. 

On physical examination, the patient's blood pressure measured 220/120 mmHg and pulse rate was regular at 64 beats/min. Clinical examination of central nervous system demonstrated pupil of equal size with normal light response, Glasgow Coma Scale (GCS) score of 14 and nystagmus in the right eye.

**Figure 1:**
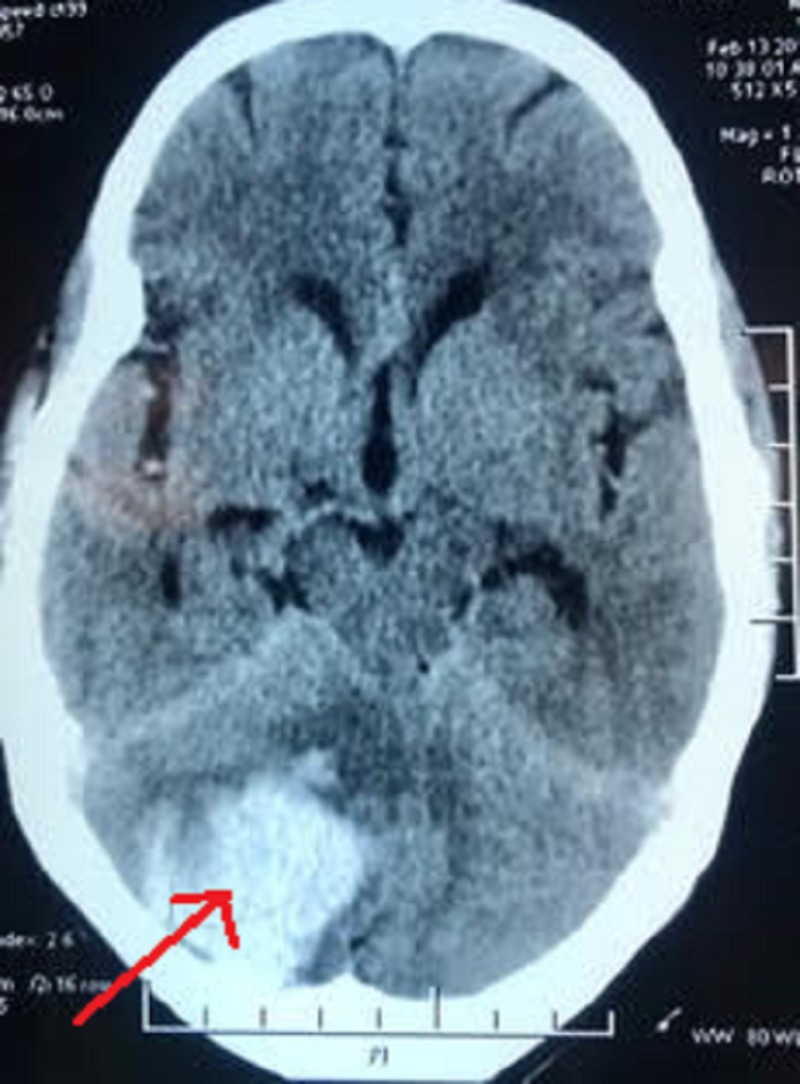
Pre-operative CT head showing right-sided cerebellar hemorrhage with effacement of fourth ventricle

Emergent CT scan of head was performed which revealed a right-sided cerebellar hemorrhage with edema, effacement of fourth ventricle and obstructive hydrocephalus (Figure 1).

Specific hematological investigations, liver function tests and coagulation profile were all within the normal range. Mannitol 20% infusion to control the intracranial pressure was started. Triple antihypertensive medications (amlodipine, losartan and prazocin) were administered to control the blood pressure.

**Figure 2:**
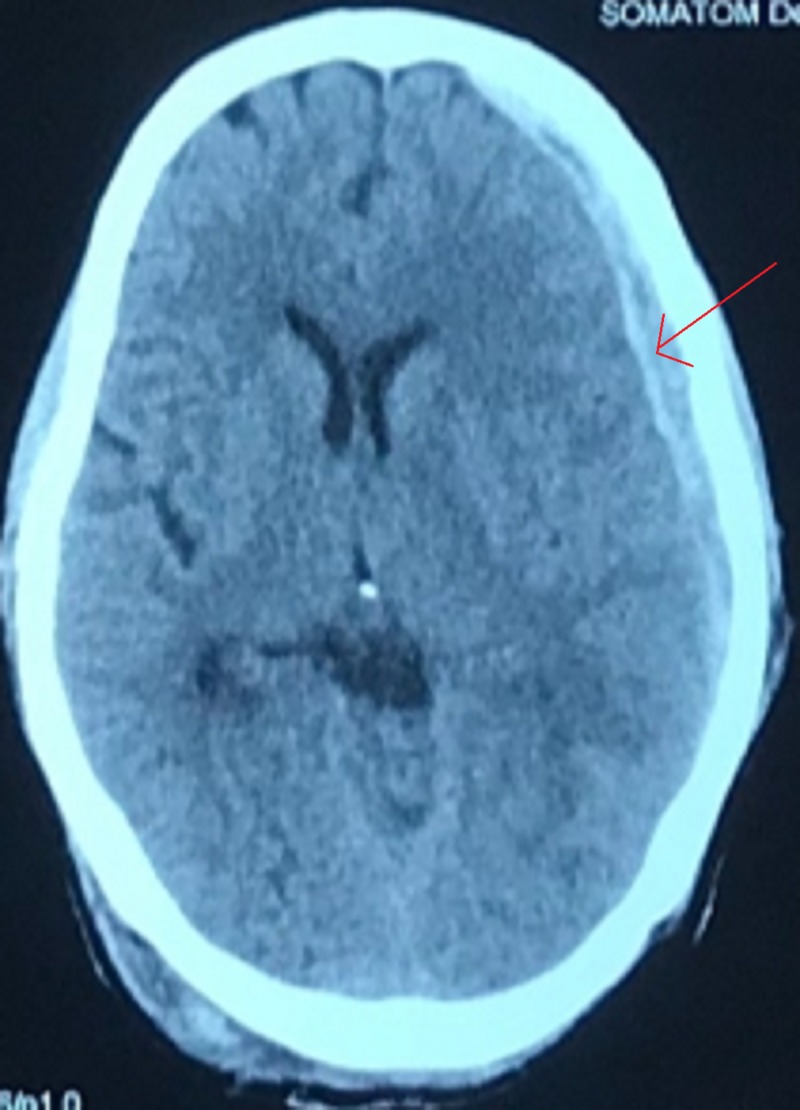
Post-operative CT scan revealing a remote contralateral (left-sided) subdural hematoma involving the fronto-parieto-temporal region

Right paramedian suboccipital craniectomy and hematoma evacuation were performed in the prone position with head in a horseshoe headrest. Intra-operatively, the dura was tight and hematoma was under pressure. After the surgery, headache and vomiting subsided and GCS score was 15. A routine repeat CT scan of head was done at six hours post surgery, which revealed a contralateral (left-sided) remote subdural hematoma involving the fronto-parieto-temporal region (Figure 2).

The patient was managed conservatively for the ASDH with decongestant (intravenous mannitol 20% for five days) and monitoring as his GCS score was 15. The patient was followed up after two weeks. During the follow-up, the patient was stable without any complaint of headache or vomiting.

## Discussion

Intracranial hemorrhage (ICH) is a devastating disease. The overall incidence of spontaneous ICH worldwide is 24.6 per 100,000 person-years with approximately 40,000 to 67,000 cases per year in the United States [8]. The most important risk factors for ICH include hypertension and cerebral amyloid angiopathy [9]. Longstanding hypertension leads to hypertensive vasculopathy causing microscopic degenerative changes in the walls of small-to-medium penetrating vessels, which is known as lipohyalinosis [10]. The commonest site for hypertensive bleed is basal ganglia (55%), followed by thalamus (26%), cerebral hemispheres (11%), brain stem (8%) and cerebellum (7%) [11]. Immediate surgical intervention is indicated in cerebellar hematoma when there is neurological deterioration, brainstem compression and/or hydrocephalus from ventricular obstruction [12].

For many years, decompressive craniectomy has been used in the management of patients with elevated intracranial pressure and cerebral edema. However, it may release the tamponade effect and result in brain shift which exposes these patients to remote ICH [13].

Four cases of supratentorial hematoma following microvascular decompression for trigeminal neuralgia were reported by Nozaki et al., among which two of the patients had same side and rest had contralateral ASDH [14]. Similarly, a case reported by Lv et al. also showed contralateral subdural hematoma following evacuation of ASDH (Table 1) [4]. However, in our case there was contralateral remote supratentorial ASDH following hematoma evacuation in a patient with hypertensive cerebellar hemorrhage.

**Table 1:** Case reports of supratentorial hematomas following decompressive craniotomy or craniectomy

Cases	Age in years	Gender	Side of hematoma	Author
1	50	Female	Contralateral	Nozaki et al. [14]
2	49	Female	Ipsilateral	Nozaki et al. [14]
3	74	Female	Contralateral	Nozaki et al. [14]
4	51	Male	Ipsilateral	Nozaki et al. [14]
5	49	Male	Contralateral	Lv et al. [4]

**Figure 3:**
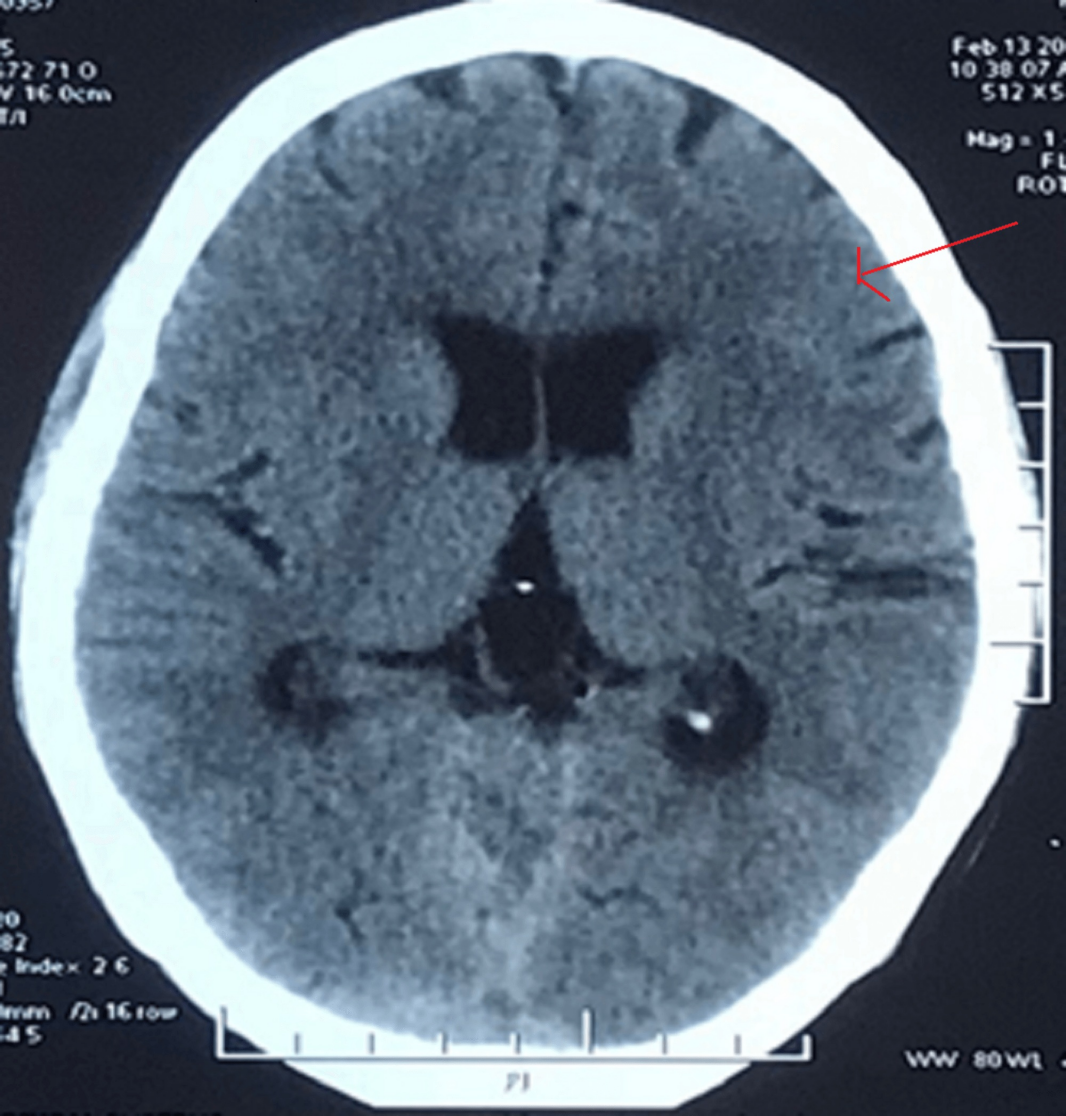
Pre-operative CT head at the frontotemporal level showing no subdural hematoma.

A case of contralateral ASDH reported by Lv et al. had slight contralateral ASDH on pre-operative cranial computed tomography [4]. We re-evaluated pre-operative CT scan of our patient to see whether we missed any supratentorial bleed, which was absent (Figure 3).

There are reports of intracranial hematoma in patients operated with head holder pins [15]. Our patient was operated in a horseshoe headrest, so this possibility could also be ruled out.

Although the exact causes of postsurgical remote hemorrhages remain unclear, the following factors should be considered: (1) excessive drainage of cerebrospinal fluid, which may cause brain distortion and laceration of the bridging veins; (2) excessive rotation and flexion of the neck, which may obstruct jugular venous drainage on the contralateral side and cause subsequent venous hemorrhagic infarction in the supratentorial regions (Hanakita and Kondo); (3) a sudden increase in cerebral blood flow with defective autoregulation, damage to cerebral vasculature secondary to perioperative parenchymal shift, previously undetected contusion and bleeding secondary to decompression and coagulopathy [16,17].

Intraoperative sonography with probe gently placed over dura can sometimes identify remote hematomas, which could lead to timely intervention and prevention of life-threatening mass effect by hematoma expansion [18]. Any unexplained features of increased intracranial pressure following hematoma evacuation warrant immediate CT scan of head keeping in mind the possibility of remote ASDH.

The natural course of ASDH is highly variable; although most of them resolve spontaneously with conservative management, few do not disappear and may require surgical intervention. Patients with GCS score >8, age <65 years and with small ASDH will have the best functional outcomes [19]. Our patient was managed conservatively as he was younger than 65 years, his GCS score was good and there was no neurological deficit.

## Conclusions

Contralateral supratentorial subdural hematoma following craniectomy and evacuation of hypertensive cerebellar hematoma is a rare, but a potentially life-threatening complication. High index of suspicion is required if patients do not improve after craniectomy and evacuation of cerebellar hematoma. This is to ensure serial neurological examinations of the patient and brain imaging if needed following craniectomy and evacuation of cerebellar hematoma for early detection of contralateral supratentorial subdural hematoma in order to decrease morbidity and mortality.
